# Uncovered diversity of infectious circular RNAs: A new paradigm for the minimal parasites?

**DOI:** 10.1038/s44298-024-00023-7

**Published:** 2024-04-18

**Authors:** Joan Marquez-Molins

**Affiliations:** Department of Plant Biology, Uppsala BioCenter, Swedish University of Agricultural Sciences and Linnean Center for Plant Biology, Uppsala, Sweden

**Keywords:** Viral evolution, Metagenomics, Viral epidemiology

## Abstract

Infectious circular RNAs (circRNAs) have been considered as biological oddities only occurring in plants, with limited exceptions. However, a great diversity of viroid-like circRNAs has been recently uncovered by the high-throughput exploration of transcriptomic data of geographically and ecologically diverse niches. In my opinion, this suggests a change in basic assumptions regarding our knowledge about these minimal parasites. The potentially infectious circRNAs found are diverse in size, type of ribozymes, encoded proteins and potential host organisms. The distinction between viroids and RNA viruses has been blurred by the detection of circular mitoviruses and ambiviruses which encode for their own RNA-dependent RNA polymerase. Thus, their taxonomic classification might pose a challenge because of the apparent extensive horizontal transfer and recombination of sequences. Many aspects of the predicted circRNAs remain to be uncovered, such as their pathogenicity or host range, and experimental validations are essential. For example, viroid-like circRNAs similar in size to plant viroids have been found to replicate and cause symptoms in fungi, with an isolate being the smallest replicon characterized so far. Despite an ancestral prebiotic origin for viroid-like sequences has been proposed, their dependence of viral or cellular proteins seems, to my view, more compatible with a cellular escape and/or viral genome reduction. This wide variety of potentially infectious agents might pose a biohazard concern of which we were previously unaware, and thus it would be convenient that more efforts are assigned for their characterization.

## Introduction

The vast majority of infectious ribonucleic acids (RNAs) have a linear genome that encodes for several proteins, as for example *Tobacco mosaic virus* which was the first virus discovered^1^ or SARS-CoV2, the causal agent of the COVID pandemic^2^. However, there are instances of infectious RNAs with a covalently-closed genome found in nature. But so far, these infectious agents have been considered as biological oddities with a restricted host range. Nevertheless, recent discoveries challenge this perspective following the discovery of a diverse collection of potentially infectious circular RNAs (circRNAs) with characteristics reminiscent of viroids^3–5^.

**Fig. 1:**
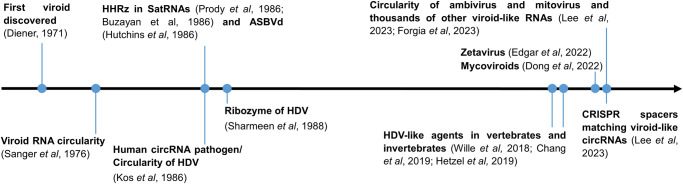
Milestones in the discovery of infectious circular RNAs. HHRz: hammerhead ribozyme, SatRNA: Satellite RNA, ASBVd: avocado sunblotch viroid, HDV: hepatitis delta virus. Mycoviroids: infectious agents of the fungus *Botryosphaeria dothidea*. Zetavirus: small viroid-like circular RNAs with paired ribozymes and open reading frames.

Discovered in the early seventies by Theodor Diener (Fig. 1), viroids were defined as low molecular weight autonomously-infectious RNAs, much smaller than any virus^6^. The circularity of their genome was observed a few years later by electronic microscopy^7^, constituting the first circRNAs discovered in nature. Viroids replicate through a rolling circle (RC) mechanism that produces longer than unit replication intermediates. These multimeric RNAs are processed into a circRNA molecules in the range of a few hundreds of nucleotides (around 300 nts). In the following years, more viroids were discovered naturally infecting higher plants and according to their structural and functional features were classified in two families: *Avsunviroidae* and *Pospiviroidae*. Members of the family *Avsunviroidae* replicate in the chloroplast and have in their sequence self-cleaving ribozymes which are essential for their replication^8^. In particular, the ribozymes present in this family are called hammerhead ribozymes (HHRz) and process the replication intermediates generated by a RC mechanism into unit length genomes. Conversely, members of the family *Pospiviroidae*, lack ribozymes, replicate in the nucleus, have a central conserved region and typically have a rod-shaped secondary structure^9^. In short, viroids are the smallest and simplest known infectious agents, causing in some cases symptomatic infections in plants with agricultural importance^10^.

In addition to viroids, other infectious circRNAs have been discovered, but lacking the capacity to infect autonomously (Fig. 1). These agents have viroid-like features like the presence of ribozymes and high degree of base pairing but in contrast are encapsidated. Those dependent of a helper virus for replication are named satellite RNAs (satRNAs)^11,12^ and they have a similar size to viroids and also infect mostly plants^13^. In contrast, an agent causing acute hepatitis in humans was found to have a larger circRNA genome (approximately 1700 nts)^14^ and replicate using the host RNA polymerase II^15,16^. This agent was named hepatitis delta virus (HDV) and encodes for an antigen, but depends on the hepatitis B virus for packaging, release, and transmission^17^. Moreover, HDV has ribozymes in each polarity termed delta ribozymes (DVRz)^18^. This was the first and only circRNA agent known in animals for more than 20 years (Fig. 1). Therefore, the identification of infectious circular RNAs had been limited to plants for decades with this sole exception of HDV as a human pathogen^19^. However, since 2018, a number of delta-like agents have been identified using transcriptomic data of diverse metazoan species^20–23^. Contrary to the human HDV, none of the newly described delta-like genomes has been found associated with a coinfecting hepadnavirus. Therefore, these infectious agents might be associated with another virus or replicate autonomously. In support of the later hypothesis, a rodent deltavirus was not found associated to any virus, and it was shown to autonomously replicate in transfected cells^24^.

Therefore, the previous paradigm assuming that HDV was the sole exception to viroids and viroid-like agents infecting plants was initially challenged by the discovery of several delta-like agents in vertebrates and invertebrates^20–23^.

### Metatranscriptome mining uncovers a great diversity of ribozyme-bearing circRNAs

Recent studies have identified a large number of putative infectious circRNAs, as a result of the increasing abundance of metatranscriptomic data^3–5^. These computational approaches were based on the identification of head-to-tail repeats (hallmarks of circularity or RC replication) and the search for ribozymes by sequence and secondary structure homologies^19^. For that, transcriptomic data of geographically and ecologically diverse niches were analyzed, finding thousands of new potentially infectious circRNAs. These findings have profound implications regarding the diversity of these minimal parasites and thus may redefine our understanding about their host range and molecular properties. Therefore, they further challenge our basic assumptions about the previous view of viroid-like RNAs as almost exclusive to plants. First, using the *Serratus* cloud computing infrastructure for petabase-scale sequence alignment against the delta antigen protein and ribozymes, hundreds of new potentially novel viruses with circular genomes and ribozymes embedded in their sequence were predicted^3^. In addition to more *bona fide* delta viruses in different animal hosts, 39 different circRNA genomes encoding the delta antigen protein but with HHRz instead of DVRz were also found^3^. These genomes together with the ones discovered in toads and termite ants that had been experimentally demonstrated to have HHRz in each polarity^25^ were named as epsilon viruses^3^. Additionally, over 300 genomes ranging from 324–789 nt were predicted to fold into rod-like structures and contain HHRz in both orientations^3^. Moreover, up to 90% of these genomes are multiple of 3 and encode two open reading frames (ORFs), one sense and one anti-sense^3^. The name zeta virus was proposed for these minimal circRNA genomes^3^, and interestingly, some plant viroids as *Hop stunt viroid* also have non-terminated ORFs in both polarities^26^. These exciting findings paved the way for exploring the diversity of potentially infectious circRNAs.

Subsequently, 5131 metatranscriptome datasets collected from diverse ecosystems were analyzed to identify viroid-like circular RNA^5^. This approach looked for any known self-cleaving ribozymes in computationally identified circRNAs. A total of 10,181 sequences unrelated to known viroid species were found and clustered into 4823 putative species when an average nucleotide identity (ANI) of 90% level was considered^5^. Of this total amount, only around 30% contained predicted ribozymes in both polarities (paired ribozymes)^5^. In another study, 32,393 circRNAs with paired ribozymes were found using *Serratus*, and clustered into 20,364 viroid-like species considering 90% of ANI^4^. These numbers would have been larger if predicted circular RNAs with only one ribozyme were considered, as unknown ribozymes might be present in the other polarity. In fact, studying these predicted circRNAs seems a promising approach to discover new ribozymes^27^. Additionally, infectious circRNAs agents lacking ribozymes such as pospiviroids might possibly exist, relying on host factors for their cleavage. Therefore, these studies are conservative in predicting the number of potential infectious circRNAs, and further approaches will likely expand these numbers. It is also noteworthy the creation of a dedicated database to viroid-like RNAs that will help with the study and classification of these agents^28^.

**Fig. 2:**
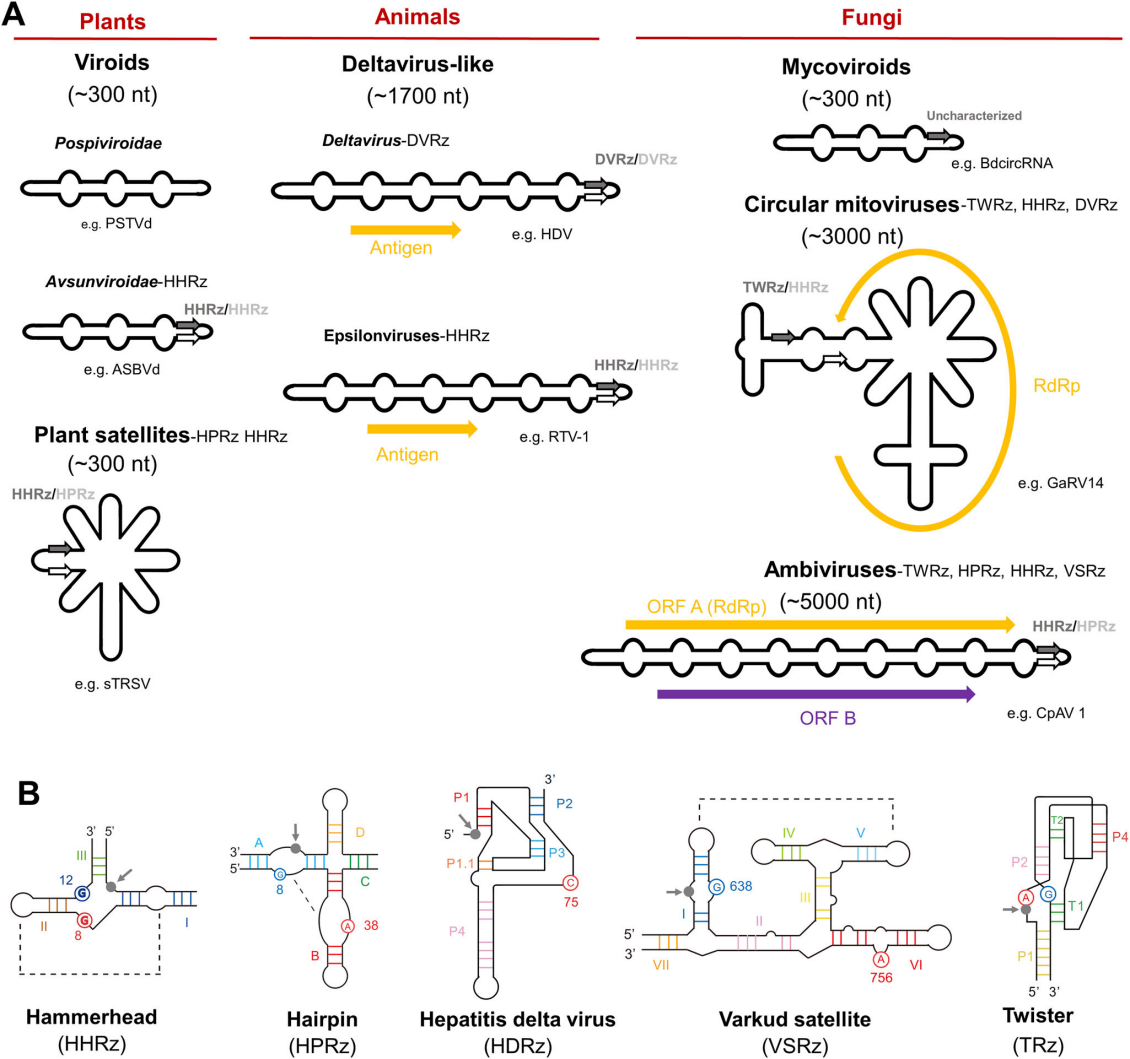
Genomic diversity of representative groups of infectious circular RNAs with assigned hosts. **A** Comparative genome organization with a representative agent of each taxonomic group. The types of ribozymes identified in each group are indicated in the right of each one. **B** Schematic secondary structure of the five types of ribozymes (Rz) validated to be active in infectious circRNAs. The approximate cleavage site is indicated with a grey dot and an arrow. The structures of the ribozymes are adapted from Ribocentre: a database of ribozymes^56^. *PSTVd* potato spindle tuber viroid, *ASBVd* avocado sunblotch viroid, *sTRSV* satellite tobacco ringspot virus, *HDV* hepatitis delta virus, *RTV-1* rhinotermitid virus, *GaRV14* grapevine associated mitovirus 14, *CpAV1* cryphonectria parasitica ambivirus 1.

The coexistence of two different ribozyme types in the genome of circRNAs was exceptionally reported only in a few circRNAs: some satRNAs having HHRz together with a hairpin ribozyme^29^ (Fig. 2A). However, in these studies^4,5^, unusual combinations of different ribozymes were frequently detected, in addition to other ribozymes not previously found in viroid-like RNAs, such as twister, Varkud Satellite (Fig. 2B) and more rarely hatchet and pistol (although neither of which were found in both strands of any circRNA). Thus, the diversity of self-cleaving ribozymes occurring in infectious circRNAs is much wider than anticipated and clearly reflects the heterogeneity of these parasitic agents (Fig. 2).

Regarding the size of predicted circRNAs genomes, most were smaller than 800 nts^4,5^, and thus in the range of plant viroids and zetaviruses. However, larger elements encoding proteins with homology to known viruses were also present. It was especially noticeable the detection that ambivirus (~5 kb) and some mitovirus (~3 kb) genomes encoding for RNA-dependent RNA polymerases (RdRp) are indeed circular^4,5^ with extensive base-pairing and contain functional ribozymes in each polarity^4^. These virus and viroid-like RNA hybrids replicate and accumulate in fungi^30^, and it was speculated that might have arisen as a result of recombination of a viral RdRp with a viroid-like genomic backbone^4^. Therefore, their taxonomic classification is unclear because of this presence of hybrid features. Since they encode for a RdRp gene they could be included in the realm *Riboviria*^31^ but the presence of ribozymes could justify their inclusion in the realm *Ribozyviria*^32^. Genomes larger than 3 kb represent only ~4% of those analyzed, while those of approximately 1 kb were ~15%^4^. Intriguingly, no infectious circRNA of around 1 kb has been characterized so far, suggesting that there might be an important group of replicative forms yet to be characterized.

In short, the vast amount of predicted viroid-like RNAs implies an unprecedented complexity in biological roles of these agents, as it was proposed in the recent review by Ma et al.^33^. This variety of potential infectious agents might pose a biohazard concern of which we were previously unaware, as it was thought that their host range was essentially restricted to plants. Therefore, characterizing the diversity, transmission and pathogenicity of these agents would be useful to evaluate their potential risks, if any. Moreover, it would be enriching to study the effects that these potential infectious agents might cause in their host and how this may contribute to their phenotypic plasticity.

### Experimental validation is required to confirm the infectivity of circRNAs

Most of the circRNAs identified were found in environmental metatranscriptomes and therefore assigning a host for these agents is difficult. In fact, only some have been assigned to stablished taxonomic groups (Fig. 2A). However, the identification of many of the circRNAs in diverse samples increases the likelihood of really being infectious agents. Nonetheless, given the frequent presence of ribozymes in DNA genomes, it cannot be excluded the possibility that they may be novel families of retrozymes, which are retrotransposons that have ribozyme sequences and propagate via circular RNA intermediates^34,35^. This is particularly pertinent for the identified circRNAs containing a ribozyme in just one polarity^5^ since all retrozymes described so far have just one self-cleaving ribozyme. Therefore, to confirm the infectivity of predicted circRNAs it would be necessary to asses that exist only as RNA forms and replicate through RC. Indeed, both metatranscriptome analysis highlight the necessity of molecular validation of these predicted circRNA agents^4,5^.

The natural host range of the less than 50 accepted viroid species was considered to be limited to higher plants. However, very recently, exogenous circRNAs with viroid-like features were isolated from the fungus *Botryosphaeria dothidea*^36^. These circRNAs were named BdcircRNAs, range in 157–450 nts in length, replicate in the nucleus of *B. dothidea* and their infection can cause symptoms. The inoculation of healthy isolates of the fungus with BdcircRNAs suggested the autonomous infection. However, caution is required in the interpretation of these results, since most mycoviruses result in asymptomatic infections^37^. Therefore, the possibility that a mycovirus, on which BdcircRNAs might be dependent, was present cannot be completely discarded yet. To confirm the autonomous replication of these proposed mycoviroids, it would have been very convenient an RNA-seq analysis of the propagated fungal cells following artificial inoculation experiments. Interestingly, despite the fact that all the BdcircRNAs sequences were phylogenetically related, only a subset contained a functional ribozyme, which was different from the ones previously described. The smallest BdcircRNA isolate is currently the smallest infectious agent described (157 nt). However it remains to be studied how stable these RNA species are considering the likely high-mutation rate during replication^38^. It was believed that the theoretical minimum replicator size should be of ~200 nt because of the viral genome reduction observed in artificial conditions favoring replication speed that generated the “Spiegelman monster” of 218 nt^39^. It may be speculated that, since these mycoviroids replicate in the nucleus, they might use the host DNA-dependent RNA polymerase II as it has been shown for pospiviroids. Thus the mutation rate would not be as high as in avsunviroids^40^. Additionally, other characterized viroid-like circRNAs with ribozymes and similar size to viroids might also have a fungal host, although this is still pure speculation and requires experimental validation^41–43^. Moreover, other satellites in the size range of viroids but with a dsRNA genome have also been proposed to infect fungi^44^. Therefore, fungi not only host hybrids with viroid-like features encoding viral proteins, but also seem to be capable of hosting minimal infectious circRNAs or true viroids, as it was recently reviewed by Sato and Suzuki^30^. Since many fungi are parasites of animal and plants, they could have been an evolutionary hub for the horizontal transfer or these agents, as it was proposed by Forgia et al.^4^.

Regarding the possibility that circRNAs infect non-eukaryotic hosts, a promising hint was the discovery of bacterial CRISPR spacers matching a cluster of circRNAs with viroid like features, such as ~300 nt size, paired HHRz and high-degree of base pairing^5^. Therefore, it will be illuminating to validate if these circRNAs can indeed replicate in a bacterial host. This would demonstrate that the host range of infectious circRNAs is much wider than previously thought and not restricted to eukaryotes.

### Complex evolutionary origins of infectious circRNAs

Viroids and viroid-like agents have been proposed to be fingerprints of the prebiotic RNA world because of their extreme simplicity and the presence of ribozymes^45–47^. However, it has been argued that the likelihood of maintaining specific sequences unchanged for billions of years is remote^48,49^. Thus, instead of direct descendants of primordial RNA replicators, it seems more plausible that these agents could have emerged from cellular genomes. There are several observations in support of this hypothesis. First, the widespread presence of ribozymes in prokaryotic and eukaryotic genomes^50,51^. There are intriguing similarities between retrozymes and avsunviroids. Both replicate through a RC process, are detected in both polarities, contain the same type III HHR motifs and are ligated (at least in vitro) by tRNA-ligases^34^. In fact, a recent study suggest that a retrozyme would be capable of replication and systemic transport in *Nicotiana benthamiana* plants^52^. Second, the requirement of sophisticated cellular or viral protein polymerases for their replication. No autoreplicative ribozyme has been identified in any of the viroid-like agents^53^. Indeed, these agents are constrained to be cellular parasites and their similarities with the predicted ancient circRNA replicators could be attributed more to convergence rather than direct evolution.

The hypothesis of a viral reduction has also been discussed^49^. Infectious circRNAs encoding RdRp such as ambiviruses and some mitoviruses could have given rise to smaller circRNAs such as delta virus and epsilon virus following the elimination of the RdRp gene. In support of this, most ambiviruses encode HHRz and some encode DVRz, while the small and simple delta antigen is highly divergent and could have been horizontally transferred by another infectious agent. The extreme case of genomic reduction could be zeta viruses which have a similar size to plant viroids but endless ORFs in each polarity. However, the function of these ORFs, if any, remains to be elucidated. Both cellular escape and viral reduction do not have to be mutually incompatible options, since escape and integration of mobile genetic elements in cellular genomes is not uncommon^54^. Indeed, the evolutionary history of circRNA infectious agents seems to be shaped by multiple recombination and horizontal transfer events. The extreme diversity of infectious circRNAs argues against a monophyletic origin, and minimal circRNA replicators could have arisen independently several times during evolution. It may be hypothesized that the self-cleaving ribozymes present in infectious circRNAs could be the true survivors of an ancient RNA world. However, it has been shown that ribozymes, such as HHRz could easily evolve de novo^55^ and thus might appear during evolution.

## Conclusions

Recently there has been a change in basic assumptions from the perspective of circRNA agents exclusively infecting plants (with the sole exception of HDV in humans), to the current situation in which they are ubiquitous in many other eukaryotes and probably even prokaryotes (Fig. 1). A great diversity of viroid-like circRNAs has been uncovered by the high-throughput exploration of transcriptomic data of geographically and ecologically diverse niches, which is expected to increase exponentially leading to new discoveries. These potentially infectious circRNAs are diverse in size, type of self-cleaving ribozymes, encoded proteins and host organisms, and only few have been assigned a host (Fig. 2). The distinction between viroids and RNA viruses has been blurred by the finding of infectious circRNAs encoding RdRp. Thus, their taxonomic classification will pose a challenge because of their foreseeable complex evolutionary relationships based on the apparent extensive horizontal transfer and recombination of sequences^4,5^. Many aspects of these potentially infectious circRNAs remain to be discovered, such as their pathogenicity or host range, and experimental validations are essential. Despite an ancestral prebiotic origin for viroid-like sequences has been proposed, a cellular escape and/or viral genome reduction could be, in my opinion, more plausible hypothesis for the emergence of the described infectious circRNAs. This is based on the widespread presence of ribozymes in prokaryotic and eukaryotic genomes and the strict dependence of viroid-like genomes on sophisticated cellular or viral protein polymerases for their replication. These minimal RNA replicons or ultimate parasites can complete their life cycle without encoding proteins by coopting viral or host proteins. Therefore, they are a prominent example of the functional versatility of RNA molecules. Moreover, all the predicted diversity of potentially infectious agents might pose a biohazard concern of which we were previously unaware, and thus it would be convenient that more efforts are assigned for their characterization.

## Data Availability

No datasets were generated or analysed during the current study.
